# Immunomodulatory effects of interferon-γ on human fetal cardiac mesenchymal stromal cells

**DOI:** 10.1186/s13287-019-1489-1

**Published:** 2019-12-04

**Authors:** Karl-Henrik Grinnemo, Marie Löfling, Lubov Nathanson, Roland Baumgartner, Daniel F. J. Ketelhuth, Vladimir Beljanski, Lindsay C. Davies, Cecilia Österholm

**Affiliations:** 1 Department of Molecular Medicine and Surgery, Karolinska Institutet, BioClinicum J10:20, SE-171 64 Solna, Sweden; 2 Division of Cardiothoracic Surgery and Anesthesiology, Department of Surgical Sciences, Uppsala University, Akademiska University Hospital, 751 85 Uppsala, Sweden; 3 Institute for Neuroimmune Medicine, Dr Kiran C. Patel College of Osteopathic Medicine, Nova Southeastern University, Davie, FL USA; 4 Department of Medicine Solna, Cardiovascular Medicine Unit, Center for Molecular Medicine, Karolinska Institutet, SE-171 64 Solna, Sweden; 5 Cell Therapy Institute, Dr Kiran C. Patel College of Allopathic Medicine, Nova Southeastern University, Davie, FL USA; 6 Department of Laboratory Medicine, Karolinska Institutet, SE-141 52 Huddinge, Sweden

**Keywords:** Mesenchymal stromal cells, Fetal hearts, ISL1, Inflammation, Immunomodulation, Programmed cell death protein 1

## Abstract

**Background:**

Mesenchymal stromal cells (MSCs), due to their regenerative and immunomodulatory properties, are therapeutically used for diseases, including heart failure. As early gestational-phase embryonic tissues exhibit extraordinary regenerative potential, fetal MSCs exposed to inflammation offer a unique opportunity to evaluate molecular mechanisms underlying preferential healing, and investigate their inherent abilities to communicate with the immune system during development. The principal aim of this study was to evaluate the effects of interferon-γ (IFNγ) on the immunomodulatory effects of first-trimester human fetal cardiac (hfc)-MSCs.

**Methods:**

hfcMSCs (gestational week 8) were exposed to IFNγ, with subsequent analysis of the whole transcriptome, based on RNA sequencing. Exploration of surface-expressed immunoregulatory mediators and modulation of T cell responses were performed by flow cytometry. Presence and activity of soluble mediators were assessed by ELISA or high-performance liquid chromatography.

**Results:**

Stimulation of hfcMSCs with IFNγ revealed significant transcriptional changes, particularly in respect to the expression of genes belonging to antigen presentation pathways, cell cycle control, and interferon signaling. Expression of immunomodulatory genes and associated functional changes, including indoleamine 2,3-dioxygenase activity, and regulation of T cell activation and proliferation via programmed cell death protein (PD)-1 and its ligands PD-L1 and PD-L2, were significantly upregulated. These immunoregulatory molecules diminished rapidly upon withdrawal of inflammatory stimulus, indicating a high degree of plasticity by hfcMSCs.

**Conclusions:**

To our knowledge, this is the first study performing a systematic evaluation of inflammatory responses and immunoregulatory properties of first-trimester cardiac tissue. In summary, our study demonstrates the dynamic responsiveness of hfcMSCs to inflammatory stimuli. Further understanding as to the immunoregulatory properties of hfcMSCs may be of benefit in the development of novel stromal cell therapeutics for cardiovascular disease.

## Introduction

Co-ordination of the immune response in pregnancy is central to ensuring fetal survival and development. The expression of allogeneic paternal antigens by the fetus and evasion of an ensuing maternal immune response has been attributed to populations of trophoblasts at the maternal-fetal interface [1]. During pregnancy, bi-directional exchange of maternal and fetal cells occurs through the circulation, so-termed maternal-fetal cellular trafficking (MFCF), commencing at week 7 of gestation with increasing levels of micro-chimerism through the pregnancy [2]. The principal role of MFCF is not fully understood, but has been reported to play roles in immune surveillance, development of the fetal immune system, tolerance, and tissue repair [3–7]. Pregnancy constitutes a challenge for the immune systems of the mother and fetus, and puts strict requirements on the fetal-maternal interface to prevent fetal rejection, while maintaining the capacity to combat infections. Although trophoblasts at the fetal-maternal interphase have been thoroughly studied in this context, the forming fetal organs and tissues have not, and knowledge regarding the capacity of developing fetal tissues to generate and modulate immune responses is very limited [8].

Mesenchymal stromal cells (MSCs) are increasingly recognized for their regenerative and anti-inflammatory properties, currently being investigated in over 900 clinical trials (www.clinicaltrials.gov search term = mesenchymal stem cells) as a potential treatment for a large variety of diseases, including inflammatory conditions, such as graft versus host disease [9]. Proposed therapeutic mechanisms of action are increasingly being unraveled, including the role of MSC licensing through their exposure to pro-inflammatory cytokines, such as interferon γ (IFNγ) [10]. Many studies have demonstrated that exposure to such cytokines modulates MSCs to an anti-inflammatory phenotype, associated with the expression of numerous immunomodulatory factors [11, 12]. IFNγ has been linked to the upregulation of indoleamine 2,3-dioxygenase (IDO) production, which results in tryptophan deprivation in the microcellular environment, as well as, the formation of bioactive metabolites [13]. Thus, IDO upregulation has been implicated in immunomodulation through induction of T cell apoptosis and antimicrobial actions [2, 14, 15]. In addition to its link to MSC licensing, IDO has also well-recognized functions in pregnancy, cancer, and cardiovascular disease [16, 17].

More recently, MSC mechanism of action has been linked to the cell surface expression and secretion of programmed death ligands (PD-L) [18]. These immunosuppressive factors bind to the PD-1 receptor on the surface of active T cells, known to secrete pro-inflammatory factors including IFNγ, tumor necrosis factor alpha (TNFα), and interleukin (IL)-2. The PD-L1:PD-1 pathway has been implicated as an important immunomodulatory pathway in fetal-maternal tolerance between PD-L1 expressing trophoblasts and PD-1-positive lymphocytes at the site of implantation [19].

As our knowledge of MSC immunomodulatory effects increases, it has become apparent that the tissue origin of the cells provides unique phenotypic cues. Species-dependent differences, with regard to predominant pathways identified, also require caution when interpreting data from animal models [13]. Therefore, a thorough understanding as to the molecular responsiveness to inflammatory signals is essential in each stromal cell population and cannot be extrapolated from findings linked to other stromal subsets. Such large variations, with regard to clinical conditions that could be treated with stromal cells, in addition to phenotypic variations based on the tissue origin of the cells, present a great challenge when implementing MSCs as a viable and reproducible treatment option.

Although IFNγ is expressed during early phases of pregnancy and has been shown to play important physiological roles in angiogenesis and endometrial vascular remodeling, fetal cells, including human fetal cardiac MSCs (hfcMSCs), may also be exposed to high levels of IFNγ due to viral infection or autoimmunity in utero [20]. As documented for the developing fetal immune system, the stages of pregnancy see a shift in the inflammatory status of the maternal-fetal environment. Immune cells of maternal origin, such as natural killer cells and activated T cells, may constitute the major sources of IFNγ production, as may fetal counterparts, which can be found as early as gestational weeks 7–8 in the thoracic region [21]. It is therefore important to elucidate the behavior of early fetal cardiac MSCs that encounter inflammation, and other insults common to those that afflict the adult heart during myocardial infarction or other cardiovascular diseases.

We have previously reported the phenotypic profile of hfcMSCs derived from first-trimester hearts [22]. The principal aim of this current study was to evaluate the responsiveness of hfcMSCs to IFNγ and delineate key downstream immunoregulatory pathways modulated by their exposure to inflammation. We have utilized RNA sequencing to obtain an in-depth and non-biased characterization of the hfcMSC transcriptome and validated our findings, using in vitro analyses to phenotypically and functionally evaluate how such stimuli modulate the behavior of first-trimester hfcMSCs. This study provides valuable information, which will aid in the identification of key, human-specific MSC properties that will further our understanding of cardiac stromal responses to inflammation; knowledge which may be further exploited for the development of novel therapies in cardiovascular disease.

## Materials and methods

### Isolation and maintenance of hfcMSCs

hfcMSCs used in the present study were isolated and characterized as previously reported, including demonstrated expression of ISL1, TBX18, and NKX2.5 [22]. The cells utilized in this study were also assessed for the expression of cell surface markers (CD105, CD90, CD73, and human leukocyte antigen [HLA] I), while being negative for HLA-DR and the hematopoietic lineage markers CD14, CD11, CD80, CD83, CD34, CD45, and endothelial marker CD31.

Briefly, the heart was pre-digested overnight at 4 °C in Hanks Balanced Salt Solution (HBSS) containing 0.5 mg/ml trypsin (ThermoFisher Scientific, Stockholm, Sweden). The mesenchymal fraction was prepared according to a modified version of a protocol developed by Laugwitz et al. [23]. The pre-digested tissue underwent several rounds of collagenase II (Worthington Biochemical Corp., NJ, USA) treatment (160 U/ml in HBSS at 37 °C with gentle stirring). The derived cell suspension was subsequently seeded onto cell culture plastic at a density of 50,000 cells/cm^2^ to recover the adherent fraction. After 96 h of culture at 37 °C in 3% CO_2_, including one medium change, the cells were detached using TrypLE™ Express (ThermoFisher Scientific), washed twice, and thereafter cryopreserved for later use.

For the below experiments, hfcMSCs at passages 4–6 were seeded at a density of 30,000 cells/cm^2^ on cell culture plastic coated with a thin layer of Geltrex™ (ThermoFisher Scientific) and maintained in DMEM/F12 supplemented with B27 (ThermoFisher Scientific), 2% (v/v) fetal bovine serum (FBS; ThermoFisher Scientific), Mycozap (Lonza Group Ltd., Basel, Switzerland), 10 ng/ml epidermal growth factor (R&D Systems, Abingdon, UK), and 100 ng/ml Wnt3a (R&D Systems).

### IFNγ stimulation of hfcMSCs

hfcMSCs were cultured ± 100 IU/ml recombinant human (rh) IFNγ (PeproTech Nordic, Stockholm, Sweden) for 2 or 7 days with media changes at day 4. Where conditioned media was to be harvested, the culture media and rhIFNγ treatment were additionally replaced 24 h prior to harvest to ensure equal conditioning times between experimental groups. Conditioned media were aspirated, centrifuged for 6 min at 230*g*, and thereafter aliquoted and stored at − 80 °C. Cells were harvested using 0.05% Trypsin-EDTA (ThermoFisher Scientific), counted for data normalization and subsequently used for flow cytometry as described below.

### RNA sequencing

Following IFNγ treatment, and culture in parallel without stimulation, hfcMSCs (*n* = 7 per group) were pelleted, lysed, and stored at − 80 °C in RLT buffer (QIAGEN AB, Sollentuna, Sweden) containing 143 mM β-mercaptoethanol (Sigma-Aldrich Sweden AB, Stockholm, Sweden). Total RNA was purified using the RNeasy Micro Kit (QIAGEN) to a final volume of 14 μl. Of this, 10 μl was used to generate cDNA using the Clontech SMARTer Kit (Takara Bio Europe AB, Gothenburg, Sweden). After library generation using the Nextera XT Kit (Illumina, Uppsala, Sweden), pooled libraries were loaded at 10 pM on High-Output flow cells onto a HiSeq2500 System (Illumina) and 50 base single-end read sequencing was performed. All procedures were performed according to the manufacturer’s instructions. Quality control assessment was carried out using an Illumina RNA-sequencing pipeline to estimate genomic coverage, percent alignment, and nucleotide quality. Raw sequencing data were transformed to fastq format. Raw reads were mapped to the reference human genome (the most recent build GRCh38) using GSNAP [24], MapSplice [25], HISAT2 [26], and STAR [27] software.

For the differential analysis of known transcript reads for each gene aligned by GSNAP, MapSplice and HISAT2 were counted using HTSeq software [28]. Alignment by STAR was run with the option “quantMode TranscriptomeSAM” that allowed counting of reads aligned to each gene. Raw counts from HTSeq and STAR were imported into Bioconductor/R package edgeR, normalized, and tested for differential gene expression [29]. This analysis was performed separately for the files produced by each aligner. In each analysis, we selected genes that were differentially expressed based on the criteria of a false discovery rate (FDR) less than 5% and a fold change more than 1.3 in either direction. Genes that showed differential expression after analysis of the files from at least two aligners were selected for further analysis as illustrated by the Venn diagram shown in the Additional file 1. A list of these genes was imported into the Ingenuity Pathway Analysis software (IPA, QIAGEN) for pathway analysis and for discovery of upstream regulators.

### Nanostring

Validation of expression of selected genes was performed as described in Additional file 2 using nCounter Elements technology (NanoString, Seattle, USA).

### hfcMSC surface marker expression with IFNγ stimulation

hfcMSCs cultured +/− rhIFNγ (*n* = 4) were detached as described above and stained with directly conjugated antibodies as outlined in Additional file 3. Cells were run on a FACSCalibur (BD Biosciences, CA, USA) with 30,000 gated events recorded, and analyzed using FlowJo Version 7.6 (BD).

### IDO metabolite quantification

Tryptophan and kynurenine concentrations in hfcMSC culture supernatants (*n* = 4) were measured by high-performance liquid chromatography (HPLC) according to Zhang et al. [30]. Briefly, 15 μl 70% (w/v) perchloric acid (Sigma-Aldrich) was added to 135 μl of culture supernatant. The mixture was vortexed thoroughly, incubated on ice for 10 min, and thereafter centrifuged at 15000*g* for 10 min at 4 °C. Subsequently, the supernatant was transferred into a fresh tube and 100 μl was injected into the HPLC for subsequent analysis. Samples were eluted using a reverse phase SUPELCOSIL™ column (C18) (Supelco®, Sigma-Aldrich), with a mobile phase of 10 mM sodium dihydrogen phosphate: methanol (73:27, v/v) at pH 2.8, and a flow rate of 1.0 ml/min at 37 °C. Tryptophan and kynurenine were detected using a Photodiode Array detector (Shimazu, Kyoto, Japan) at 220 nm and 362 nm, respectively. Calibration curves for tryptophan and L-kynurenine (both from Sigma-Aldrich) were established by injecting standard solutions at different concentrations.

### Assessing the effects of hfcMSCs on the viability, activation, and proliferation of T cells

Peripheral blood mononuclear cells (PBMCs) were isolated from buffy coats by centrifugation on Ficoll-Isopaque (Lymphoprep™, Abbott Diagnostics Technologies AS, Oslo, Norway), and untouched CD3^+^ T cells were isolated by magnetic activated cell sorting (MACS; Human Pan T Cell Isolation Kit; Miltenyi Biotec Norden AB, Lund, Sweden) as previously described [18]. Where cell proliferation was assessed, PBMCs were incubated with 0.25 μM CellTrace™ CFSE (ThermoFisher Scientific) for 7 min at 37 °C. The reaction was quenched by the addition of 3× volumes of FBS and the cells washed 3 times in RPMI 1640 medium supplemented with penicillin (100 U/ml), streptomycin (0.1 mg/ml), l-glutamine (2 mM; ThermoFisher Scientific), and 10% heat-inactivated pooled human blood type AB serum (T cell media). Stained PBMCs were rested for 20 min at 37 °C before setting up the experiment. Proliferation data are expressed as a proliferation index. This value represents the total number of T cell divisions divided by the number of cells that underwent at least one division.

hfcMSCs (passages 4–5; *n* = 4) were seeded onto plates coated with Geltrex™. T cells were activated using anti-CD2/CD3/CD28 microbeads (Miltenyi Biotec) at a 1:2 bead to cell ratio and cultured in T cell media at a 1:10 ratio to hfcMSCs, either in direct contact or in 0.4 μm polyethylene terephthalate transwell membrane inserts. Where relevant, anti-PD-1 (2.45 μg/ml monoclonal mouse anti-human PD-1, #329911; Biolegend®, CA, USA) was added to co-cultures. Cells were co-cultured for 3 days. PBMCs/T cells were subsequently stained with anti-CD3 V450 (#560365; BD), anti-CD4 PerCP-Cy™5.5 (#560650; BD), anti-CD25 PE (#555432; BD), and anti-PD-1 eVolve™655 (#86-2799; eBioscience™, ThermoFisher Scientific) to assess T cell activation and proliferation, or anti-CD3 V450 in combination with Annexin V PE (#640907; Biolegend®) and LIVE/DEAD® Fixable Far Red Dead Stain Kit (#L10120; ThermoFisher Scientific) to assess viability. Samples were run on a BD LSRFortessa™ (BD), with 50,000 gated events recorded per sample and analyzed using FlowJo Version 7.6.

### Evaluating the hyporesponsive state of T cells induced by hfcMSCs

T cells from the above transwell co-cultures were counted and transferred to 96-well plates (1 × 10^5^ cells/well) in fresh T cell media ± fresh activation microbeads. Where appropriate, 15 ng/ml IL-2 (PeproTech) was added to cultures to evaluate potential for reverting to a responsive state as previously described [18]. Cells were maintained for 3 days before adding 5 Ci/mM ^3^H thymidine and culturing for a further 16 h at 37 °C. Cultures were harvested onto a glass fiber filter (Harvester 96, Tomtec Inc., CT, USA) and radioactivity quantified using a micro-β scintillation counter (PerkinElmer Sverige AB, Upplands Väsby, Sweden).

### Enzyme-linked immunosorbent assay (ELISA)

Secretion of soluble (s)PD-L1 and sPD-L2 was measured using ELISA as per the manufacturer’s instructions (R&D Systems). For sPD-L1 measurement, conditioned media samples were concentrated approximately 2.5-fold (degree of concentration measured for each individual sample) using 10 K cut-off Amicon® Ultra centrifugal filters (Millipore AB, Solna, Sweden).

### Statistics

Comparisons were statistically analyzed using Student’s *t* test or Mann-Whitney *U* test where data did not fulfill requirements for parametric testing (normal distribution and equal variances). Significance was assumed at *P* < 0.05 (Prism 5.0; Graphpad Software Inc., CA, USA and SPSS Statistics 24.0: IBM, NY, USA).

## Results

### hfcMSCs express increased levels of IFNγ receptor 1 and human leukocyte antigens upon prolonged exposure to IFNγ

**Fig. 1:**
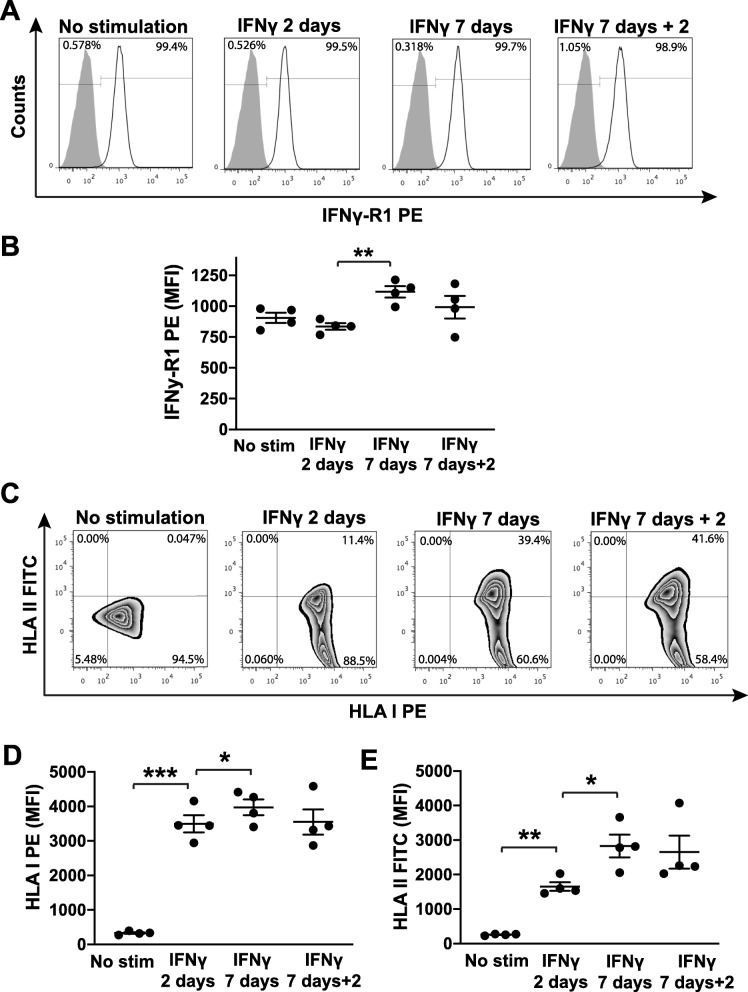
Expression of cell-surface receptors upon stimulation with IFNγ. **a** Histograms showing antibody staining of cell surface-expressed IFNγ-R1 (colored white) on hfcMSCs from one representative donor compared to IgG1 PE-negative controls (colored gray). IFNγ-R1 was constitutively expressed on all hfcMSCs, with no change in the frequency of positive cells with IFNγ exposure. **b** Scatter dot plot showing median fluorescence intensity (MFI) of IFNγ-R1 surface expression from cell cultures using four different donors. Bar indicates mean values ± standard error of the mean (SEM). The significantly increased expression remained after removal of IFNγ. **c** Density plots showing simultaneous staining of HLA I and HLA II molecules on the cell surface of hfcMSCs from one representative donor. Expression of HLA II was induced upon IFNγ stimulation and further increased after 7 days of stimulation. **d** MFI values ±SEM of HLA I expression are depicted in the scatter dot plot. **e** MFI values ±SEM of HLA II expression are depicted in the scatter dot plot. Both HLA types remained upregulated at 2 days after withdrawal of IFNγ. Independent experiments were carried out in triplicates, each time using an *n* = 4 donors. *P < 0.05, **P < 0.01, ***P < 0.001

IFNγ is one of the predominantly occurring cytokines during inflammation, and an established licensing factor for MSCs [10, 31]. hfcMSCs were cultured up to 7 days in the presence of IFNγ to establish the effects of licensing the cells to an anti-inflammatory phenotype. Flow cytometry analysis confirmed that hfcMSCs constitutively express IFNγ-receptor (R)1 (CD119; Fig. 1a) and that the level of expression, as assessed by median fluorescence intensity (MFI), significantly increased with prolonged, 7-day, exposure to IFNγ (Fig. 1b; IFNγ 2 days vs IFNγ 7 days; *P* < 0.01). Likewise, HLA I was constitutively expressed on the surface of the hfcMSCs (Fig. 1c), with significant upregulation in MFI after 2 days of IFNγ stimulation (Fig. 1d; *P* < 0.001), and furthermore at day 7 (Fig. 1d; *P* < 0.05). These elevated levels of cell surface expression of HLA I were maintained after removal of the IFNγ and further culturing of the cells in basal media for 48 h (Fig. 1d). HLA II was not present on the surface of unstimulated hfcMSCs (Fig. 1c), with a low percentage of cells switching on HLA II after 2 days of stimulation (Fig. 1c), accompanied by a significant increase in MFI (Fig. 1e; *P* < 0.01). Approximately half of the cells upregulated HLA II after 7 days of IFNγ treatment (Fig. 1c), with a further increase in MFI (Fig. 1e; *P* < 0.05), which was maintained after removal of IFNγ and continued culturing in basal media (Fig. 1e). These results are consistent with previous findings indicating that fetal cells may possess different degrees of inertia with regard to induced expression of HLA class II molecules [32]. Based on our current results, we decided to use a 7-day exposure to IFNγ in subsequent experiments.

### IFNγ alters the gene expression profile of hfcMSCs

**Fig. 2:**
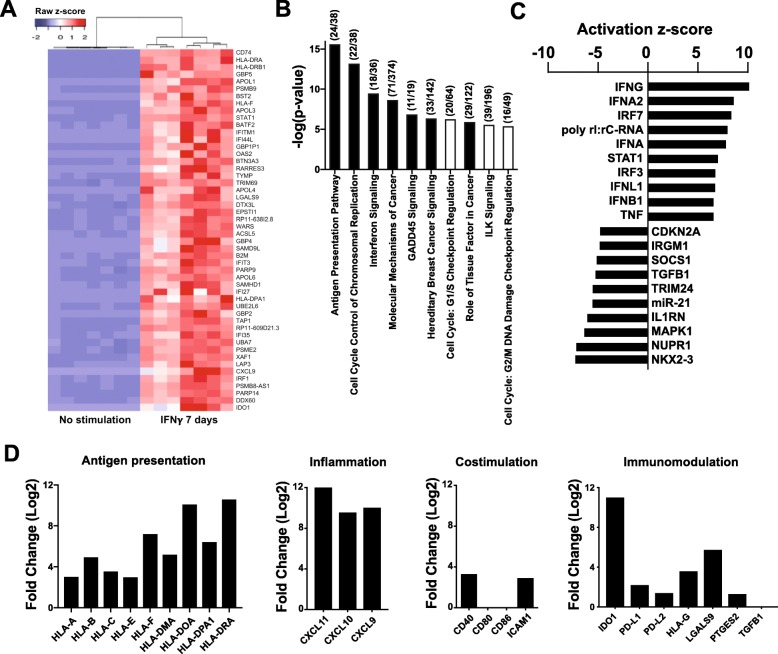
Gene expression analysis of hfcMSCs treated with IFNγ for 7 days. **a** Effects on the hfcMSC transcriptome by IFNγ stimulation (*n* = 7 donors in each group), shown as a heatmap depicting the 50 most upregulated genes. **b** Functional characterization of differentially expressed genes following IFNγ treatment based on Ingenuity Pathway Analysis (IPA). Bar height refers to the number and magnitude of differentially expressed genes in each pathway; black color indicates a positive *z*-score for the pathway, whereas white indicates a negative *z*-score, which are calculated based on the expected contribution from up- or downregulated genes. The numbers in brackets refer to (number of differentially expressed genes/number of genes assigned to a particular pathway in IPA). **c** The bar graph depicts the top ten positive and top ten negative predicted regulators of differential gene expression from the data set. **d** Upregulation of genes of interest identified from RNA sequencing data, where the respective biological functions of the selected genes are indicated on top of each bar graph. The fold changes depicted in the bar graphs are generated by analysis of difference between the two groups using STAR aligner. A summary of the corrected *P* values (FDR) for the different genes are presented in table form in Additional file 2

To determine transcriptional responses to inflammatory stimuli, hfcMSCs were cultured in the presence or absence of IFNγ for 7 days, prior to RNA sequencing. Gene expression levels were corroborated by Nanostring analyses on selected genes (Additional file 2). Unsupervised clustering analysis revealed two distinct gene clusters: IFNγ-treated and untreated, displayed as a heat map (Fig. 2a). Canonical pathway analysis primarily identified differential expression of genes belonging to antigen presentation pathways, cell cycle control, and interferon signaling (Fig. 2b). Analysis of pathway interconnection revealed pro-inflammatory pathways linked by increases in antigen presentation class I and II molecules (Additional file 4), and interferon-inducible genes (Additional file 5). We performed upstream analysis to identify key transcriptional regulators linked to the major changes in gene expression seen, in order to illuminate biological activities occurring in hfcMSCs stimulated with IFNγ. These analyses are based on the expected effects using the IPA [33]. The top activators and inhibitors are formulated from the observed gene expression profiles. This analysis predicted positive activation *z*-scores for upstream regulators, such as cytokines IFNγ, IFNα2, IFNλ1, IFNβ1, and TNF, and transcription factors, such as interferon regulatory factors (IRF) 3 and 7, as well as signal transducer and activator of transcription (STAT) 1, all of which were predicted to regulate cellular responses to IFNγ treatment. Conversely, transcriptional regulators NK2 homeobox 3, nuclear protein 1, tripartite motif containing 24, cyclin-dependent kinase inhibitor (CDKN) 2A, cytokine interleukin 1 receptor antagonist (IL1RN), microRNA (miR)-21, transforming growth factor (TGF) β1, and immune-related GTPase family M member 1, as well as, suppressor of cytokine signaling 1, constitute the top 10 predicted inhibitors (Fig. 2c).

IFNγ treatment also induced pro-inflammatory chemokines, such as C-X-C motif chemokine ligand (CXCL)9, CXCL10, and CXCL11 and several immunomodulatory genes, including IDO1, PD-L1 (B7-H1), PD-L2 (B7-DC), HLA-G, and galectin-9 (LGALS9). However, no increase in expression of antigen presentation-associated co-stimulatory molecules such as CD80 (B7-1) and CD86 (B7-2) could be observed, with the exception of CD40 and Intracellular Adhesion Molecule-1 (ICAM-1), which were slightly elevated (Fig. 2d).

### Altered gene expression upon IFNγ stimulation correlates with upregulated expression of surface-expressed and secreted modulators of immune responses

Based on the most differentially expressed genes extracted from our transcriptomics data, we chose to assess protein levels of PD-L1/PD-L2 and enzymatic activity of IDO.

**Fig. 3:**
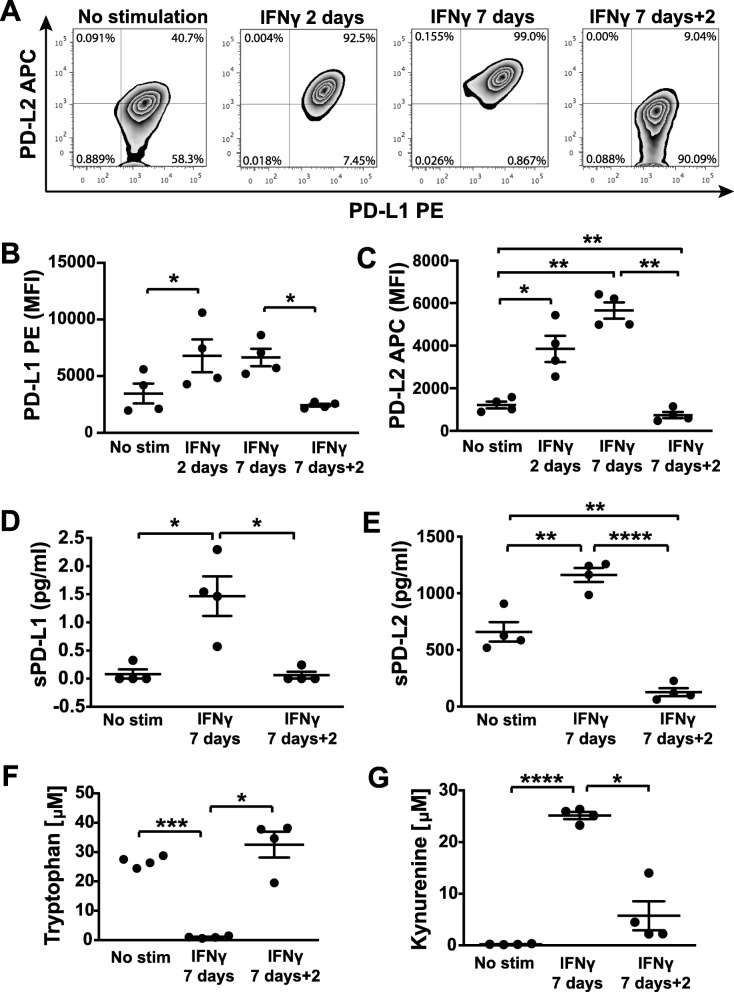
Surface-expressed and soluble immunoregulatory mediators upregulated by hfcMSCs following IFNγ stimulation. **a** Density plots showing dual staining of cell surface expressed co-inhibitory receptors PD-L1 and PD-L2. **b** Scatter dot plots showing median fluorescence intensity (MFI; bar indicates mean ± standard error of the mean [SEM]) for surface-expressed PD-L1 and **c** PD-L2, which were both constitutively expressed in non-stimulated hfcMSCs, and increased significantly in response to IFNγ stimulation, but dropped to background levels or below after removal of the cytokine. **d** Soluble (s)PD-L1 was detected in the media after 7 days of IFNγ stimulation, whereas **e** detectable levels of sPD-L2 were secreted from resting hfcMSCs. An increase in sPD-L2 could be measured after IFNγ stimulation. Both sPD-L1 and sPD-L2 dropped back to or below background levels after withdrawal of the pro-inflammatory stimulus. **f** Upregulation of the IDO1 gene was reflected in an increased consumption of tryptophan shown as reduced levels, in addition to **g** elevated levels of the metabolite kynurenine, serving as a surrogate marker for IDO expression and activity. This was observed in hfcMSCs from all donors analyzed, as demonstrated by the kynurenine to tryptophan ratios. IDO activity rapidly returned to baseline levels upon withdrawal of IFNγ, shown as retained tryptophan levels and concomitantly reduced kynurenine levels 2 days after media replacement. The experiments were performed on cells from four different donors (*n* = 4). Graph data are presented as mean ± SEM. *P < 0.05, **P < 0.01, ***P < 0.001, ****P < 0.0001

The cell surface levels of immunomodulatory molecules PD-L1 and PD-L2 were evaluated in unstimulated and IFNγ-stimulated hfcMSCs. hfcMSCs constitutively expressed cell surface PD-L1, with a subset of the population also positive for PD-L2 at resting state (Fig. 3a, mean number of 59% positive cells ± 16% standard deviation [SD]). After 2 days of stimulation with IFNγ, the proportion of PD-L2-positive cells increased compared to unstimulated controls (Fig. 3a, mean 96% ± 3% SD). This was maintained at 7 days of stimulation (Fig. 3a, mean 98% ± 0.45% SD). Removal of IFNγ and further culturing for 48 h significantly reduced the number of PD-L2 positive hfcMSCs back to equivalent numbers seen within the unstimulated controls (Fig. 3a, mean 30% ± 20% SD). Although the hfcMSCs were constitutively positive for PD-L1 expression, the MFI significantly increased with exposure to IFNγ for 2 days (Fig. 3b, *P* < 0.05), with comparable intensity seen after 7 days of stimulation, but a drop back to the MFI of unstimulated controls after removal of the IFNγ (Fig. 3b, *P* < 0.05). A similar trend was demonstrated for PD-L2, with significant increases in intensity seen with 2 days and 7 days of IFNγ stimulation compared to controls (Fig. 3c, IFNγ 2 days *P* < 0.05 and IFNγ 7 days *P* < 0.01). Interestingly, after removal of IFNγ, MFI dropped to a level significantly lower than the base level intensity seen in the unstimulated controls (Fig. 3c, *P* < 0.01).

We also evaluated how IFNγ stimulation modulates hfcMSC secretion of sPD-L1 and sPD-L2. Secretion of both sPD-L1 and sPD-L2 was significantly upregulated in response to IFNγ stimulation (Fig. 3d, *P* < 0.05 and Fig. 3e, *P* < 0.01). The concentrations of sPD-L2 were substantially higher than those measured for sPD-L1, both in resting and IFNγ-stimulated hfcMSCs. Consistent with the data obtained for cell surface expression, the secreted levels of both sPD-L1 and sPD-L2 dropped to or below baseline levels upon removal of IFNγ (Fig. 3d, *P* < 0.05 and Fig. 3e, *P* < 0.0001).

As IDO was one of the most upregulated genes identified by the transcriptomics analysis, we assessed its activity in response to IFNγ stimulation. The tryptophan levels were equal in the unstimulated cell cultures from the different donors with an almost complete abolishment after 7 days of IFNγ stimulation (Fig. 3f, *P* < 0.001). Removal of the inflammatory stimulus restored the tryptophan levels back to those of the unstimulated cells, measured after 48 h (Fig. 3f, *P* < 0.05). Corresponding levels of the tryptophan metabolite kynurenine were inversely correlated, with barely detectable levels in unstimulated cells and 100-fold increased concentrations after 7 days of IFNγ stimulation (Fig. 3g, *P* < 0.0001). Although detectable after withdrawal of the inflammatory stimulus, the kynurenine levels were significantly lower than those measured from the stimulated cells (Fig. 3g, *P* < 0.05).

In order to better characterize the cells with regard to expression of co-stimulatory vs co-inhibitory molecules, we also analyzed cell surface expression of CD80, CD86, and CD40, of which the latter was slightly upregulated at the mRNA level. No significant increase could be observed, in terms of cell surface expression (as assessed by MFI), for any of the three co-stimulatory receptors (Additional file 6).

### hfcMSCs exert immunomodulatory activity, suppressing T cell activation and proliferation

**Fig. 4:**
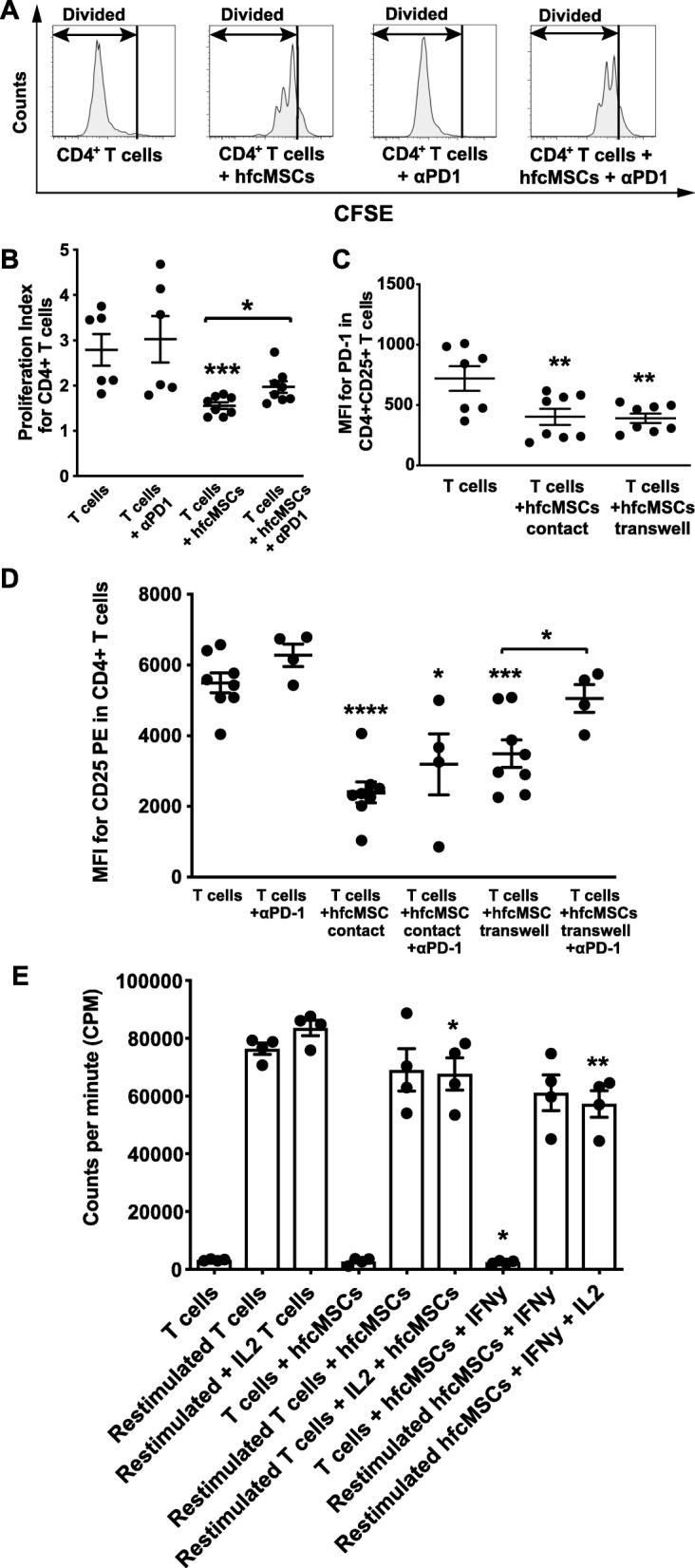
hfcMSCs exhibit immunomodulatory effects on CD4^+^ lymphocytes through regulated PD-1 expression and interaction. **a** Histograms showing CellTrace™ CFSE dilution in a representative sample of CD4+ T cells following 3 days of incubation in the presence or absence of hfcMSCs and/or blocking αPD-1 antibody. **b** The effect of hfcMSCs on CD4+ T cell division, shown as proliferation index, after co-culture with hfcMSCs in the presence or absence of blocking αPD-1 antibody. **c** The presence of hfcMSCs decreased the median fluorescence intensity (MFI) cell surface expression of PD-1 on isolated, activated CD4+CD25+ T cells. The same effect was observed when the cell types were separated by a transwell filter, indicating a predominant role for soluble mediators. **d** Effects of hfcMSCs on CD4+ T cell activation, depending on cell/cell contact or soluble mediators, shown as IL-2RA (CD25) expression after 3 days of direct co-culture or separation of the two cell types with transwell filters, in the presence or absence of blocking αPD-1 antibody. **e** The combined bar graph/scatter dot plot depicts T cell proliferation as incorporation of ^3^H-thymidine after direct co-culture with hfcMSCs, followed by restimulation with IL-2. The experiments were performed on cells from four different hfcMSC donors (*n* = 4). Data from **a**–**d** were derived from two T cell donors (*n* = 8). Graph data are presented as mean ± SEM. *P < 0.05, **P < 0.01, ***P < 0.001, ****P < 0.0001

To assess impact on CD4+ T cell proliferation, hfcMSCs were co-cultured for 5 days with PBMCs. Co-culture of PBMCs with hfcMSCs in the presence of activating anti-CD2, CD3, and CD28 microbeads suppressed CD4+ T cell proliferation, as assessed by measurement of the proliferation index (Fig. 4a, b, *P* < 0.001). Blockade of the co-inhibitory T cell receptor PD-1 significantly attenuated the suppressive effects of hfcMSCs, partially restoring the proliferation of CD4+ T cells (Fig. 4a, b, *P* < 0.05). To investigate the direct effects of hfcMSCs on the activation status of CD4+ T cells, as assessed by PD-1 and CD25 expression, hfcMSCs were cultured in direct contact or transwell systems with activated, purified CD3+ T cells. hfcMSCs in both systems suppressed the expression of PD-1 on the surface of CD4+ T cells, indicating a direct suppressive effect by the hfcMSC secretome (Fig. 4c, *P* < 0.01). Furthermore, hfcMSCs suppressed cell surface CD25 expression on CD4+ T cells in both contact and transwell systems (Fig. 4d, contact *P* < 0.0001; transwell *P* < 0.001). Blockade of the PD-1 receptor significantly restored CD25 expression on T cells co-cultured with hfcMSCs in transwell, but not in contact (Fig. 4d; *P* < 0.05). After removal of the hfcMSCs and their secretome, T cells derived from the above transwell co-cultures were restimulated in the presence or absence of IL-2, to assess T cell responsiveness [18]. Only T cells with prior exposure to hfcMSCs primed with IFNγ demonstrated a significantly suppressed baseline proliferative potential after removal of the hfcMSCs (Fig. 4e; *P* < 0.05). T cells co-cultured with hfcMSCs (+/− IFNγ priming) demonstrated no impairment in their ability to proliferate in response to restimulation (Fig. 4e). Where T cells were additionally stimulated with IL-2, a significant decrease in proliferation compared to their respective controls could be seen in cells pre-exposed to hfcMSCs (+/− IFNγ priming; Fig. 4e; *P* < 0.05 and *P* < 0.01 respectively).

### Direct contact between hfcMSCs and T cells induces cell death

**Fig. 5:**
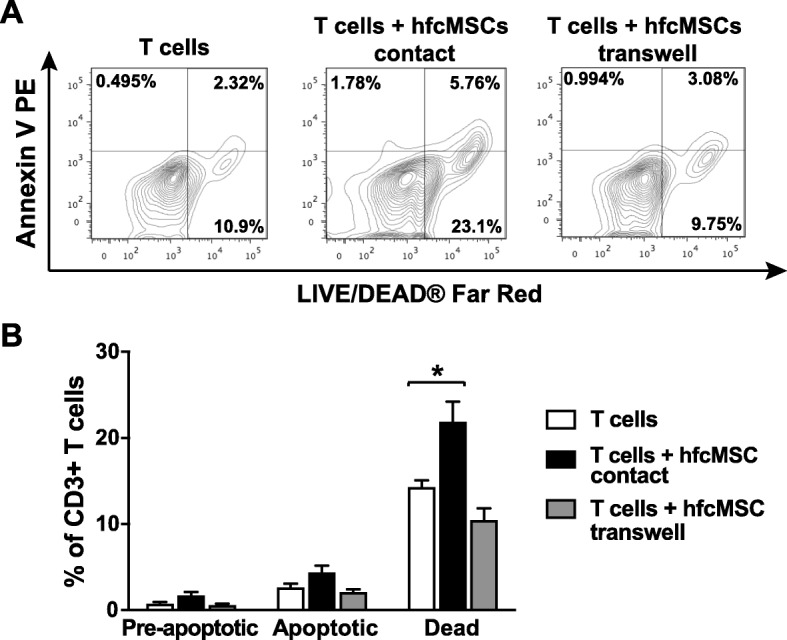
hfcMSCs induce T cell death via contact-dependent mechanisms. Levels of apoptosis and total cell death were assessed in CD3+ T cells co-cultured with hfcMSCs in contact and transwell systems. **a** Representative contour plots demonstrating the shift in CD3+ T cell viability and percentage of pre-apoptotic (Annexin V+ LIVE/DEAD®-), apoptotic (Annexin V+ LIVE DEAD®+), and total dead (LIVE DEAD®+) cells in control, contact, and transwell co-cultures. **b** Bar graph illustrating average percentage pre-apoptotic, apoptotic, and dead CD3^+^ T cells ± SEM (*n* = 4 hfcMSC donors). *P < 0.05

T cells co-cultured in direct contact or in transwell systems with hfcMSCs were assessed for the expression of the apoptosis-associated marker, Annexin V, in conjunction with a LIVE/DEAD® marker by flow cytometry (Fig. 5a). Analysis revealed no significant change in the level of pre-apoptotic (Av+LD−) or apoptotic (Av+LD+) T cells after exposure to hfcMSCs in either experimental setup (Fig. 5b). Significantly higher levels of total cell death, considering both necrotic and apoptotic fractions, were seen after exposure of the T cells to hfcMSCs in direct contact, but not in transwell co-cultures (Fig. 5b, *P* < 0.05).

## Discussion

Human fetal MSCs have been isolated and characterized from a number of sources including the liver and bone marrow [22, 34–36]. The current study reports, for the first time, a detailed mechanistic analysis of phenotypic and functional responses to inflammatory stimuli by hfcMSCs derived from first-trimester gestation, a time when the fetus still exhibits scarless wound healing and thus regenerative properties. We demonstrate that, upon IFNγ stimulation, hfcMSCs display a number of common responses with regard to the repertoire of genes regulated at the transcriptional level, compared to different adult sources of MSCs [37–39]. The data presented indicate that key modes of immunomodulation overlap between hfcMSCs and MSCs derived from other anatomical sources, but that distinct tissue- and age-specific differences are apparent [18, 40]. These data emphasize the need to delineate between stromal cell sources and establish the individual phenotype or function of this source of MSCs in their own right.

The beneficial effects of MSCs as a treatment, for a variety of conditions in clinical and experimental settings, are well documented. The mode of action of MSC-mediated modulation of the immune system remains poorly understood and seems to principally differ upon mode of cell delivery [41, 42]. Although mechanisms whereby MSCs contribute to the regeneration of diseased and injured tissue, when delivered exogenously, are being increasingly understood, the precise functions of tissue-resident MSCs can only be extrapolated. There is, however, strong evidence suggesting that licensing of the cells by exposure to pro-inflammatory signals such as IFNγ forms an integral role in conveying their immunomodulatory potential [10, 31, 43, 44].

Flow cytometry analysis confirmed that culture-expanded hfcMSCs constitutively express IFNγR1, also known as CD119, on their cell surface and are therefore primed to respond to such inflammatory signals. Furthermore, prolonged exposure to IFNγ induced further upregulation of CD119 expression, suggesting a positive feedback mechanism on the hfcMSCs. IFNγ binding to CD119 has been eloquently shown to induce downstream signaling through the JAK/STAT1 pathway leading to transcriptional upregulation of both HLA class I and II expression [45]. Here we demonstrate that hfcMSCs, like all nucleated cells, constitutively express HLA class I on their cell surface and that exposure to IFNγ upregulates this at both the gene and protein level. HLA class II is not expressed on the cell surface and, as previously shown for both fetal MSCs, and some subsets of primitive adult progenitor cells, requires prolonged (7-day) exposure to the cytokine in order to induce expression on the cell surface [32, 46].

Analysis of the transcriptome demonstrated a distinct molecular profile of hfcMSCs upon IFNγ stimulation, with clustering in antigen presentation, cell cycle control, and interferon signaling pathways. Increased mRNA expression of other immunomodulatory factors, involved in the recruitment and modulation of immune cell functions, were also observed. These findings suggest that hfcMSCs may be able to respond to inflammation and modulate their environmental milieu through cell surface expression and secretion of multiple factors. Further investigation of these transcriptional changes, in particular hfcMSC secretion of immunomodulatory factors, was conducted at the protein level and further validated using functional assays. hfcMSCs demonstrated a high degree of plasticity and quick response to environmental changes. This was evidenced by an immediate shutdown of IDO activity upon removal of IFNγ stimulation, which was accompanied with a likewise prompt downregulation of cell surface-expressed and secreted forms of PD-L1 and PD-L2. The isolated and analyzed hfcMSCs comprise the whole stromal fraction of the fetal heart. Although these cells fulfill the criteria outlined by the International Society for Cellular Therapy (ISCT) for MSCs, based on expressed surface markers and differentiation capacity [22], they still represent a heterogenous population of stromal cells. The homogenous and swift response to the addition and withdrawal of inflammatory stimuli, with low donor-to-donor variability, indicates that hfcMSCs may function to fine-tune immune responses of the fetal cardiac tissue.

Despite the shift in hfcMSC phenotype upon removal of inflammation, elevated cell surface levels of HLA classes I and II were maintained. These data indicate that the cells have the capacity to remember prior stimuli and alter their phenotype based on these environmental changes. The concept of epigenetic memory within stem cell populations is well described, especially with respect to induced pluripotent stem cells. This study validates the theory that cells can alter their genetic makeup to remember their origin and the environmental conditions that they have been previously exposed to [47]. Additionally, several reports have been published demonstrating that stress-inducing stimuli during fetal and neonatal periods can contribute to later predisposition to conditions such as type II diabetes and heart damage [48, 49].

Based on the induced expression of HLA class II genes, MSCs have been implicated as conditional antigen-presenting cells during syngeneic immune responses [50]. RNA sequencing data supported this theory as an upregulation of CD40 was seen in response to IFNγ treatment. However, flow cytometry confirmed that this transcriptional change did not result in a significant induction in cell surface expression of the CD40 protein. These findings demonstrate an additional distinct difference between hfcMSCs and adult stromal cell sources, such as BMMSCs, the latter previously having been reported to induce cell surface expression of CD40 with pro-inflammatory stimulation [51].

Functional immunomodulation experiments confirmed that hfcMSCs are able to directly suppress the activation and proliferation of T cells. The use of blocking antibodies confirmed that, like BMMSCs, hfcMSCs can downregulate expression of CD25 (IL-2RA) and T cell proliferation via PD-L1 and PD-L2 [18]. However, unlike BMMSCs [18], no induction of T cell anergy was observed, with removal of the hfcMSCs and restimulation of the T cells triggering proliferation once more. The knock-on effect of CD25 suppression by prior exposure of the T cells to hfcMSCs was evident, with IL-2 stimulation significantly dulled.

Our analysis also confirmed a significant upregulation in IDO activity, a modulator of T cell proliferation and activity through regulation of tryptophan, an essential amino acid for T cell function and survival. As previously published for more primitive progenitor cell subsets, such as oral progenitor cells, no effect on T cell apoptosis was seen with exposure to the hfcMSC secretome, suggesting that IDO does not exert a principal role in hfcMSC modulation of T cell viability [52]. In contrast, direct contact between hfcMSCs and T cells did induce a significant increase in total cell death. These findings may be indicative of stronger effects exerted by both PD-1 ligands (cell surface and secreted forms) and IDO in close proximity to the hfcMSCs, or alternatively evidence of additional immunomodulatory mechanisms employed by the hfcMSCs.

Within adult MSC populations, it has been extensively reported that MSCs can modulate the monocyte phenotype towards that of an IL-10 producing anti-inflammatory monocyte or/and macrophage. It is through this modulatory effect that MSC populations are proposed to induce tolerance and promote regulatory T cell differentiation [53]. Reports within the literature strongly indicate that fetal cells may also be able to modulate immune responses through targeting of the innate immune compartment. Second-trimester cardiac MSCs have been reported to reduce T cell proliferation, whilst promoting increased levels of TGFβ1 and IL-10 [36, 54]. It would be therefore interesting to further investigate the bystander effects of hfcMSCs, in addition to the direct immunomodulatory effects demonstrated in this study, in order to understand their breadth of potential in orchestrating the adaptive immune response.

## Conclusions

In summary, we demonstrate, for the first time, that MSCs derived from human fetal hearts of the first trimester acquire an immunomodulatory phenotype upon exposure to IFNγ. The heart is the first organ to develop in the human embryo, and the genetic pathways responding to inflammation before the fetal immune system is developed are, as we show within this study, highly conserved. Our findings hence underline the role of genetics rather than conditioning and environmental exposure in governing the major mechanisms of tolerance, inflammatory response, and tissue repair within the stroma. Exposure of hfcMSCs to a pro-inflammatory environment of this kind induced significant changes at the transcriptional level. Upregulation of a number of immunomodulatory genes corresponded with a phenotypic and functional shift in the hfcMSCs, resulting in changes such as increased IDO activity and regulation of T cell activation and proliferation. These responses, including a high degree of plasticity by the hfcMSCs, which has previously not been described, may reflect the requirements of the dynamic fetal environment and intrinsic regenerative properties. Our findings support the knowledge that, in utero, human fetal stromal cells need to respond, efficiently and appropriately, to inflammatory insults occurring during infections and other tissue injuries, while maintaining an environment that supports tissue growth and co-existence with the maternal immune system. Further studies on hfcMSCs with the aim to elucidate anti-inflammatory and associated anti-fibrotic properties will expand our knowledge of key regenerative mechanisms for future therapeutic development in the areas of cardiovascular repair and wound healing.

## Supplementary information

**Additional file 1.** Generation of gene dataset for further analysis. Venn Diagram illustrating numbers of genes with differential expression after alignment using GSNAP, MapSplice, HISAT2 and STAR.

**Additional file 2.** RNAseq statistics and Nanostring validation. **Table S1**, showing corrected *P* values (FDR) for fold-changes of mRNA expression of genes of interest. **Table S2**, showing genes selected for validation of RNA sequencing by Nanostring. Experimental procedure for Nanostring is included.

**Additional file 3.** Antibodies. List of additional antibodies used for flow cytometry characterization of surface-expressed markers.

**Additional file 4.** Antigen presentation signaling pathway. Predominant signaling pathway generated by Ingenuity Pathway Analysis (Antigen presentation).

**Additional file 5.** Interferon-response signaling pathway. Predominant signaling pathway generated by Ingenuity Pathway Analysis (Interferon-response signaling cascade).

**Additional file 6.** Flow cytometry. Surface expression of costimulatory molecules, analysed by flow cytometry.

## Data Availability

The datasets generated and/or analyzed during the current study are available from the corresponding author on reasonable request.

## Abbreviations

BMMSC: Bone marrow-derived MSC
CXCL: C-X-C motif chemokine ligand
ELISA: Enzyme-linked immunosorbent assay
FBS: Fetal bovine serum
HBSS: Hank’s Buffered Saline Solution
hfcMSCs: Human fetal cardiac mesenchymal stromal cells
HLA: Human leukocyte antigen
HPLC: High-performance liquid chromatography
IDO: Indoleamine 2,3-dioxygenase
IFNγ-R: Interferon gamma receptor
IL: Interleukin
IPA: Ingenuity Pathway Analysis
MFCF: Maternal-fetal cellular trafficking
MFI: Median fluorescence intensity
MSCs: Mesenchymal stromal cells
PBMC: Peripheral blood mononuclear cell
PD-1: Programmed cell death protein-1
(s) PD-L1/2: [Soluble] programmed cell death protein-1 ligand
Rh: Recombinant human
SD: Standard deviation
TGFβ1: Transforming growth factor beta 1
TNFα: Tumor necrosis factor alpha
