# Supplementary material for: Immunomodulatory effects of interferon-γ on human fetal cardiac mesenchymal stromal cells
Source: Stem Cell Res Ther. 2019 Dec 4;10:371. doi: 10.1186/s13287-019-1489-1 (PMC6894330; doi:10.1186/s13287-019-1489-1)

Additional file 1.

Gene expression analysis

Venn diagram showing upregulated genes identified by the four different aligners used. Genes identified as upregulated in at least 2 out of the 4 aligners were considered to qualify for further quantitative analyses of gene expression.

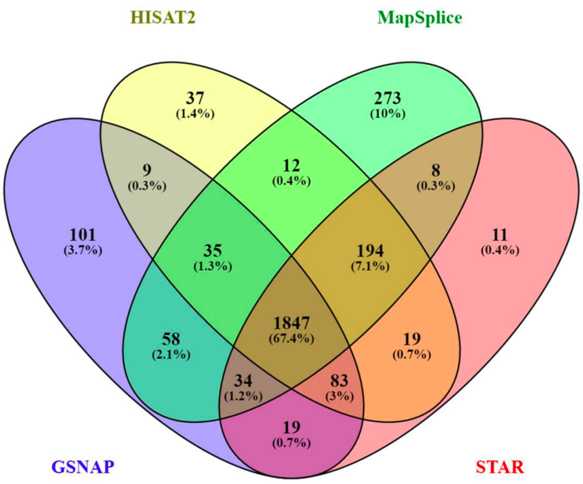

Supplement: Supplementary file 1 — Additional file 1. Generation of gene dataset for further analysis. Venn Diagram illustrating numbers of genes with differential expression after alignment using GSNAP, MapSplice, HISAT2 and STAR. [file 13287_2019_1489_MOESM1_ESM.pdf]
