# Supplementary material for: Immunomodulatory effects of interferon-γ on human fetal cardiac mesenchymal stromal cells
Source: Stem Cell Res Ther. 2019 Dec 4;10:371. doi: 10.1186/s13287-019-1489-1 (PMC6894330; doi:10.1186/s13287-019-1489-1)
Supplement: Supplementary file 2 — Additional file 2. RNAseq statistics and Nanostring validation. Table S1, showing corrected P values (FDR) for fold-changes of mRNA expression of genes of interest. Table S2, showing genes selected for validation of RNA sequencing by Nanostring. Experimental procedure for Nanostring is included. [file 13287_2019_1489_MOESM2_ESM.pdf]

Additional file 2.

Table 1. Corrected P values (FDR) for fold-changes of mRNA of genes of interest. Related to Figure 2E.

Antigen presentation		Inflammation		Costimulation		Immunomodulation	
**Gene**	**FDR**	**Gene**	**FDR**	**Gene**	**FDR**	**Gene**	**FDR**
**HLA-A**	$3.64x10^{-75}$	CXCL11	$6.05x10^{-77}$	CD40	$1.48x10^{-41}$	IDO-1	$3.91x10^{-86}$
**HLA-B**	$5.58x10^{-40}$	CXCL10	$3.57x10^{-77}$	CD80	-	PD-L1	$1.51x10^{-14}$
**HLA-C**	$5.02x10^{-32}$	CXCL9	$1.68x10^{-92}$	CD86	-	PD-L2	$1.80x10^{-9}$
**HLA-E**	$4.61x10^{-57}$			ICAM1	$2.46x10^{-9}$	HLA-G	$2.51x10^{-42}$
**HLA-F**	$4.90x10^{-189}$					LGALS9	$3.43x10^{-118}$
**HLA-DMA**	$1.66x10^{-82}$					PTGES2	0.0186
**HLA-DOA**	$8.45x10^{-56}$					TGFB1	-
**HLA-DPA1**	$3.94x10^{-103}$						
**HLA-DRA**	$2.83x10^{-303}$						

Table 2. Validation of RNA sequencing by Nanostring on selected genes. Related to Figure 2E.

	Log2 fold change	std error (log	Lower confid	Upper confid	Linear fold ch	Lower confid	Upper confid	P-value	BH.p.value	method	probe.ID		FC_G	FC_H	FC_M	FC_S
HLA-DRA	11	0,5	10	12	2080	1060	4110	8,23E-10	2,88E-08	lm.nb	NM_019111.3:335	HLA-DRA	1567,7	1512,1	1577,9	1560,9
HLA-F	10,6	0,598	9,39	11,7	1520	673	3420	7,22E-09	7,08E-08	lm.nb	NM_001098479.1:575	HLA-F	101,5	38,5	20,5	150,0
LGALS9	3,75	0,226	3,3	4,19	13,4	9,87	18,2	1,35E-08	9,44E-08	lm.nb	NM_002308.3:1206	LGALS9	55,4	54,7	49,7	52,5
CXCL11	11	0,732	9,57	12,4	2060	761	5560	3,43E-08	1,66E-07	lm.nb	NM_005409.4:282	CXCL11	5729,9	5278,3	1120,1	5713,3
IDO1	10,5	0,705	9,12	11,9	1450	555	3770	3,75E-08	1,66E-07	lm.nb	NM_002164.3:50	IDO1	2057,3	1904,3	2050,2	2046,0
CXCL9	10,8	0,73	9,39	12,3	1810	672	4890	3,93E-08	1,66E-07	lm.nb	NM_002416.1:1975	CXCL9	1048,0	1027,7	1052,3	1040,6
OAS2	9,1	0,62	7,89	10,3	551	237	1280	4,28E-08	1,66E-07	lm.nb	NM_016817.2:480	OAS2	364,5	414,0	405,6	378,3
HLA-DPA1	6,25	0,463	5,34	7,16	76,2	40,6	143	9,67E-08	2,42E-07	lm.nb	NM_033554.2:857	HLA-DPA1	86,8	94,0	89,5	87,2
HLA-DMA	6,22	0,492	5,26	7,19	74,6	38,3	146	1,78E-07	4,15E-07	lm.nb	NM_006120.3:380	HLA-DMA	34,6	34,9	35,0	34,4
DDX60	3,96	0,316	3,34	4,58	15,6	10,1	24	1,95E-07	4,26E-07	lm.nb	NM_017631.5:660	DDX60	18,0	18,9	18,6	17,9
CXCL10	3,32	0,276	2,78	3,86	9,97	6,86	14,5	2,85E-07	5,87E-07	lm.nb	NM_001565.2:461	CXCL10	756,3	974,5	763,4	751,9
OAS1	8,19	0,707	6,81	9,58	292	112	763	4,05E-07	7,87E-07	lm.nb	NM_001032409.1:805	OAS1	433,5	410,4	434,4	430,2
HLA-G	3,14	0,292	2,57	3,71	8,84	5,94	13,1	7,97E-07	1,33E-06	lm.nb	NM_002127.4:1180	HLA-G	20,4	missing	12,6	12,3
HLA-E	2,7	0,254	2,2	3,2	6,49	4,59	9,16	9,25E-07	1,47E-06	lm.nb	NM_005516.4:1204	HLA-E	6,7	6,7	8,4	6,7
IRF1	4,55	0,437	3,69	5,41	23,4	12,9	42,4	1,11E-06	1,69E-06	lm.nb	NM_002198.1:510	IRF1	27,4	28,6	28,0	27,3
IFIH1	3,94	0,419	3,12	4,77	15,4	8,71	27,2	2,78E-06	4,05E-06	lm.nb	NM_022168.2:185	IFIH1	60,7	59,1	60,8	59,7
OAS3	5,01	0,563	3,91	6,12	32,3	15	69,5	4,57E-06	6,40E-06	lm.nb	NM_006187.2:4980	OAS3	25,6	26,2	26,0	25,6
CD40	2,73	0,414	1,92	3,54	6,63	3,78	11,6	6,12E-05	7,94E-05	lm.nb	NM_001250.4:196	CD40	8,7	9,2	9,1	8,7
DDX58	2,06	0,358	1,36	2,76	4,17	2,56	6,78	0,000185	0,000231	lm.nb	NM_014314.3:2130	DDX58	missing	4,6	4,6	4,5
CD274	2,16	0,509	1,16	3,16	4,47	2,24	8,93	0,00171	0,00207	lm.nb	NM_014143.3:49	CD274	missing	4,6	4,7	4,6
PDCD1LG2	1,22	0,327	0,575	1,86	2,32	1,49	3,62	0,004	0,00467	lm.nb	NM_025239.3:235	PDCD1LG2	missing	2,6	2,6	2,6
IRF8	3,47	1,36	0,793	6,14	11,1	1,73	70,6	0,0317	0,0357	Wald	NM_002163.2:253	IRF8	478,4	452,5	472,8	473,3

RNAseq data (yellow)
Fold change by Nanostring (green)

Nanostring
Experimental procedure

nCounter Elements allows users to combine nCounter Elements General Purpose Reagents (GPRs) with unlabelled probes (listed in Table S1). 100 ng of total RNA from each sample was used to hybridize with the nCounter Elements TagSet at 67°C for 16 hours. The TagSet consists of a reporter tag and capture tag that hybridize to the user designed gene-specific probe A and probe B complex. After hybridization, the samples were washed and immobilized to a cartridge using the NanoString nCounter Prep Station. Cartridges were scanned in the nCounter Digital Analyzer at 280 fields of view for the high level of sensitivity. Positive NanoString spike-in controls and 6 highly invariant genes (SAR1B, YWHAB, SF1, ETFA, SPEN and SEC24C) served as internal controls for normalization between samples.
