# Supplementary material for: Immunomodulatory effects of interferon-γ on human fetal cardiac mesenchymal stromal cells
Source: Stem Cell Res Ther. 2019 Dec 4;10:371. doi: 10.1186/s13287-019-1489-1 (PMC6894330; doi:10.1186/s13287-019-1489-1)
Supplement: Supplementary file 3 — Additional file 3. Antibodies. List of additional antibodies used for flow cytometry characterization of surface-expressed markers. [file 13287_2019_1489_MOESM3_ESM.pdf]

Additional file 3.

Flow cytometry for characterization of cell surface markers

Assessment of cell surface-expressed markers on resting and IFNγ-stimulated hfcMSCs was performed using the following antibodies

Antibody	Fluorochrome	Product Number	Company
Mouse-anti-human CD119	PE	558934	BD Pharmingen
Mouse-anti-human HLA-ABC	PE	R7000	Dako
Mouse-anti-human HLA-DP-DQ-DR	FITC	F0817	Dako
Mouse-anti-human CD80	FITC	560926	BD Pharmingen
Mouse-anti-human CD86	FITC	560958	BD Pharmingen
Mouse-anti-human CD40	PE	560963	BD Pharmingen
Mouse-anti-human CD274 (PD-L1)	PE	12-5983	eBioscience
Mouse-anti-human CD273 (PD-L2)	APC	17-5888	eBioscience
Mouse IgG1, κ Isotype Control	APC	555751	BD Pharmingen
Mouse IgG1, κ Isotype Control	PE	551436	BD Pharmingen
Mouse IgG1, κ Isotype Control	FITC	554679	BD Pharmingen
