# Supplementary material for: Immunomodulatory effects of interferon-γ on human fetal cardiac mesenchymal stromal cells
Source: Stem Cell Res Ther. 2019 Dec 4;10:371. doi: 10.1186/s13287-019-1489-1 (PMC6894330; doi:10.1186/s13287-019-1489-1)
Supplement: Supplementary file 4 — Additional file 4. Antigen presentation signaling pathway. Predominant signaling pathway generated by Ingenuity Pathway Analysis (Antigen presentation). [file 13287_2019_1489_MOESM4_ESM.pdf]

Additional file 4.

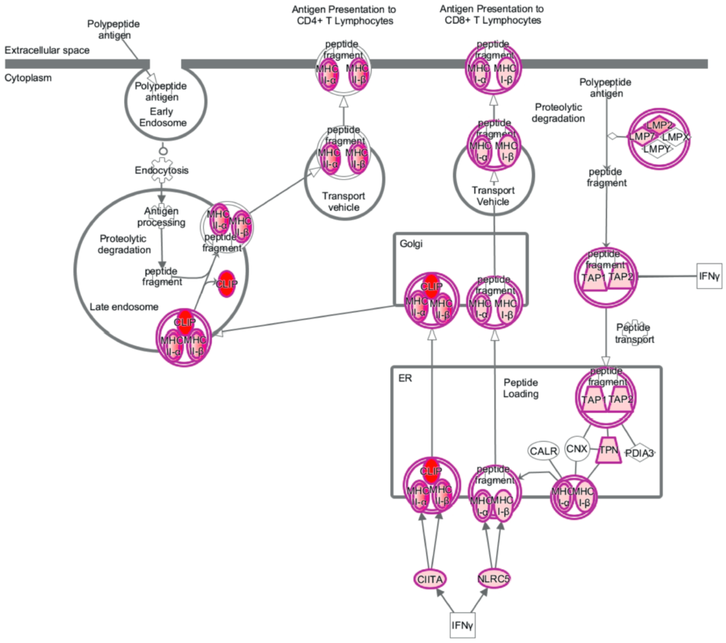

Antigen presentation signaling pathway generated by Ingenuity Pathway Analysis. Related to Figure 2
The figure shows the major signaling pathway induced by IFN-dependent upregulation of HLA class I and II genes, including cellular localization of the signaling events.
