# Supplementary material for: Immunomodulatory effects of interferon-γ on human fetal cardiac mesenchymal stromal cells
Source: Stem Cell Res Ther. 2019 Dec 4;10:371. doi: 10.1186/s13287-019-1489-1 (PMC6894330; doi:10.1186/s13287-019-1489-1)
Supplement: Supplementary file 5 — Additional file 5. Interferon-response signaling pathway. Predominant signaling pathway generated by Ingenuity Pathway Analysis (Interferon-response signaling cascade). [file 13287_2019_1489_MOESM5_ESM.pdf]

Additional file 5.

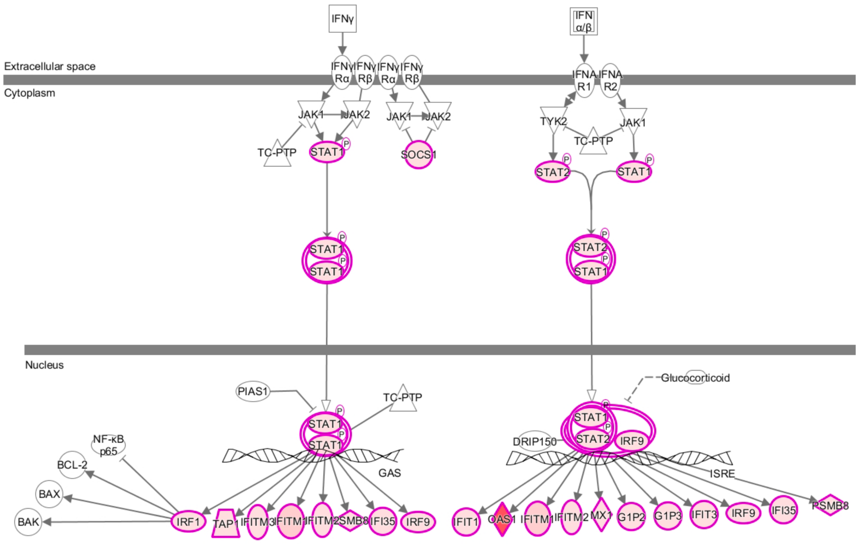

Pathways generated by Ingenuity Pathway Analysis. Related to Figure 2.

The figure shows the interferon-response signaling cascade induced in hfcMSCs upon IFNγ stimulation, where transcription of a number of interferon-inducible genes was mediated by JAK/STAT signaling.
