# Supplementary material for: Immunomodulatory effects of interferon-γ on human fetal cardiac mesenchymal stromal cells
Source: Stem Cell Res Ther. 2019 Dec 4;10:371. doi: 10.1186/s13287-019-1489-1 (PMC6894330; doi:10.1186/s13287-019-1489-1)
Supplement: Supplementary file 6 — Additional file 6. Flow cytometry. Surface expression of costimulatory molecules, analysed by flow cytometry. [file 13287_2019_1489_MOESM6_ESM.pdf]

**Additional file 6.**

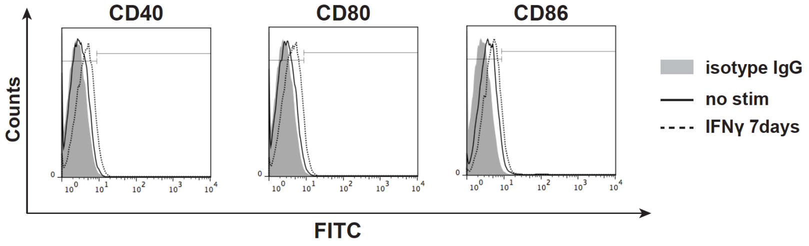


**Surface expression of costimulatory molecules. Related to Figures 2 and 3.**
No significant expression of CD80 or CD86 could be detected in resting or IFNγ-stimulated hfcMSCs, which corroborates the absence of expression at the mRNA level. Although the mRNA expression of CD40 was increased upon IFNγ stimulation, no significant corresponding upregulation of surface expressed protein could be detected.
